# The Three Conventions

**Published:** 1893-04

**Authors:** 


### Editorial.

### THE THREE CONVENTIONS.

The year 1893 promises to be a memorable one in the history of dentistry and medicine, if all the proposed conventions are carried to a successful issue. In any event the professional feeling will be stirred as never before in the same period of time.

The importance of these efforts cannot well be overestimated, and they mark an epoch in professional thought that may well claim the serious attention of all interested in progress.

The question whether these will be productive in great results or otherwise does not enter into the calculation. That they will have a marked effect there can be no question; but whether this will be noticeable, there will still be the important fact remaining, that three conventions have met, composed of various nationalities, to consider profound scientific problems. This, of itself, is a most promising outlook. It will level prejudice, broaden thought, contract geographical lines, and bring the professional ideas of the world more in harmony. At no time in the world's history has such a unifying process been attempted, or at no former period has there been conditions warranting such an effort. Language has always been a bar to international progress, and to some extent this will remain; but the levelling influences which have been at work for the past half-century have made practicable that which previously would have been impossible.

The only fear that presents itself at this time is the possibility that the work has been overdone. If time demonstrates that three conventions can be held within a month of each other, and at widely separated points, and be sustained both in regard to quality and in numbers, it will be as remarkable as anything else in this exhibition year.

From present indications this will be the result. The effort to make that at Rome equal to former meetings of the International Congress of Medicine gives promise that it will not be inferior to those that have preceded it. Whether the section of "Odontology" will do as well as in former years must depend largely upon the efforts of our European confrères, as it is not probable that many from this side will be able to meet them. The demands of the World's Columbian Dental Congress and the Pan-American Medical Congress will absorb the efforts of the large majority of the workers in America; but with the whole of Europe to draw upon, there should be a meeting in Italy nowise inferior to those heretofore held.

The outlook for the two important convocations at Chicago and Washington is satisfactory. There has been, necessarily, some friction; but this will be lessened as there is a better comprehension of facts. Some of the mistakes will, doubtless, be corrected. It this be not possible, there can be at least an earnest effort to make the former a success in spite of errors of judgment.

We have tried to impress upon the dental profession, in former articles, the importance of setting aside all predilections, and uniting on this single effort, regardless of this or that man, but solely in the interest of the profession of which we are all a part. If this be done without reserve, then will its work redound to the honor of the dentists of this country. We cannot afford to permit personal prejudices or selfish bickerings to intrude their injurious influence. They must be laid aside and the labor be taken up unselfishly, sacrificing personal feelings upon the altar dedicated to the highest good of the entire body. In this spirit alone can we hope to succeed.

The Pan-American Medical Congress is to dentistry an incident. It was conceived, as a whole, in the proper spirit; but whether it were wise to bring it in competition with the larger convention at Rome may be a question. But be this as it may, it will be held, and there is apparently no reason why the "Oral and Dental Section" should not be an entire success. The financial drain which the

Chicago Exhibition will make upon those who will feel forced to be there will, doubtless, have an effect to prevent many from visiting Washington; but there must be a large residue who will divide their interest between these two places. While it may not compare in numbers with either of the other two conventions, there is no reason why the quality of the work should be in any degree inferior. All of the foreign contingent will desire to visit Washington, and this in itself should insure a good meeting. The season during which it is held is generally delightful, and the city of Washington is a perpetual exhibition, besides being one of the most beautiful of the cities of the country.

The untiring energy which has been manifested in preparing the preliminary work of these meetings will, we are assured, find no faltering as the time rapidly lessens before the gavel calls to order. If this period be devoted to earnest work, there is no reason why September should not close with the feeling that the professional labor of 1893 has been well done, and another step taken in the onward march of ideas.

